# Decreased Cerebellar-Orbitofrontal Connectivity Correlates with Stuttering Severity: Whole-Brain Functional and Structural Connectivity Associations with Persistent Developmental Stuttering

**DOI:** 10.3389/fnhum.2016.00190

**Published:** 2016-05-03

**Authors:** Kevin R. Sitek, Shanqing Cai, Deryk S. Beal, Joseph S. Perkell, Frank H. Guenther, Satrajit S. Ghosh

**Affiliations:** ^1^Program in Speech and Hearing Bioscience and Technology, Division of Medical Sciences, Harvard Medical SchoolBoston, MA, USA; ^2^McGovern Institute for Brain Research, Massachusetts Institute of TechnologyCambridge, MA, USA; ^3^Research Laboratory of Electronics, Massachusetts Institute of TechnologyCambridge, MA, USA; ^4^Department of Speech, Language and Hearing Sciences, Sargent College of Health and Rehabilitation Sciences, Boston UniversityBoston, MA, USA; ^5^Bloorview Research Institute, Holland Bloorview Kids Rehabilitation HospitalToronto, ON, Canada; ^6^Department of Speech-Language Pathology, Faculty of Medicine, University of TorontoToronto, ON, Canada; ^7^Department of Otology and Laryngology, Harvard Medical SchoolBoston, MA, USA

**Keywords:** persistent developmental stuttering, MRI, resting state, diffusion, connectivity

## Abstract

Persistent developmental stuttering is characterized by speech production disfluency and affects 1% of adults. The degree of impairment varies widely across individuals and the neural mechanisms underlying the disorder and this variability remain poorly understood. Here we elucidate compensatory mechanisms related to this variability in impairment using whole-brain functional and white matter connectivity analyses in persistent developmental stuttering. We found that people who stutter had stronger functional connectivity between cerebellum and thalamus than people with fluent speech, while stutterers with the least severe symptoms had greater functional connectivity between left cerebellum and left orbitofrontal cortex (OFC). Additionally, people who stutter had decreased functional and white matter connectivity among the perisylvian auditory, motor, and speech planning regions compared to typical speakers, but greater functional connectivity between the right basal ganglia and bilateral temporal auditory regions. Structurally, disfluency ratings were negatively correlated with white matter connections to left perisylvian regions and to the brain stem. Overall, we found increased connectivity among subcortical and reward network structures in people who stutter compared to controls. These connections were negatively correlated with stuttering severity, suggesting the involvement of cerebellum and OFC may underlie successful compensatory mechanisms by more fluent stutterers.

## Introduction

Persistent developmental stuttering is characterized by disfluency of speech, particularly repetition or prolongation of specific sounds or parts of words such that a speaker’s ability to verbally communicate is disrupted. Over 5% of children but only 1% of adults are estimated to experience stuttering (Yairi and Ambrose, 1999; Mansson, 2000; Reilly et al., 2009). Thus, while some people recover from the speech impairment through therapy or ongoing maturation, others continue to be affected by disfluencies. Understanding how the neural patterns of people with mild stuttering compensate for their symptoms is crucial for understanding the disorder and could lead to new therapies for people with more severe stuttering.

What structural and connectivity differences lead to stuttering in the first place? While limited so far, research involving children who stutter has revealed decreased bilateral gray matter volume in frontal and temporal gyri associated with speech production (Chang et al., 2008; Beal et al., 2013). Using resting state fMRI functional connectivity and diffusion MRI structural connectivity in children who stutter, a later study found decreased whole-brain connectivity with left putamen and left supplementary area (Chang and Zhu, 2013).

To investigate compensatory mechanisms for stuttering, researchers can measure brain differences after participating in a speech therapy regimen. One such study found increased cerebellar activity during reading following a therapy intervention (De Nil et al., 2001). A different group identified increased activations in right frontal and bilateral superior temporal cortex and putamen in PWS during an overt reading task, with right frontal lobe increases continuing for at least 2 years post-training (Neumann et al., 2003; Preibisch et al., 2003). Lu et al. (2012) saw changes in resting state cerebellar activity in Mandarin speakers after a seven-day therapy intervention. Orbitofrontal regions may also enable recovery from stuttering symptoms. While right orbitofrontal cortex (OFC) is likely recruited in recovered PWS after fluency therapy, left OFC may enable PWS to overcome stuttering symptoms without therapeutic assistance (Kell et al., 2009). An MEG case study of a PWS found that left OFC activity decreased prior to a blocking event compared to a successfully produced utterance (Sowman et al., 2012).

The increase in cerebellar activity following speech fluency therapy could rely on the cerebello-thalamo-cortical pathway that is active in normal speech production (Jürgens, 2002). The cerebellum likely plays a key role in timing control of motor outputs (Stein and Glickstein, 1992; Howell, 2004). The dual-route model of motor planning suggests that a lateral pathway involving the cerebellum and premotor cortex, in contrast to the automatized basal ganglia-supplementary motor medial pathway, incorporates external stimuli and can be modulated by attention and cognitive control (Goldberg, 1985, 1991; Alm, 2004, 2005). Such a cerebello-cortical circuit could function as a compensatory mechanism for the dysfunctional basal ganglia-cortical route (Alm, 2004; Smits-Bandstra and De Nil, 2007). Indeed, as mentioned previously, speech fluency training increases cerebellar activity during reading and alters resting state cerebellar connectivity (De Nil et al., 2001; Lu et al., 2012). In stuttering, the cerebellum is typically more active during speech and is more connected with cortical networks (Lu et al., 2009, 2010). The cerebellum could compensate for diminished connections between cortical speech regions by increasing attention-driven monitoring of speech output (Allen et al., 1997; Craig-McQuaide et al., 2014), which aligns with the repeated finding of hyperactive cerebellum in stuttering (Brown et al., 2005) and with the DIVA model of speech production (Guenther et al., 2006; Civier et al., 2010; Tourville and Guenther, 2011). In the DIVA model, the cerebellum plays multiple roles in feedback and feedforward speech motor control, notably in mapping between sensory states and motor production (Tourville and Guenther, 2011). By sitting between sensory and motor representations of speech production, the cerebellum may counteract a dysfunctional primary production network by providing an additional layer of control for speech motor output.

Both cortical and subcortical mechanisms have thus been linked to persistent developmental stuttering as well as to overcoming stuttering symptoms. The aim of this study was to characterize the differences in cortico-subcortical structural and functional connectivity in PWS and persons with fluent speech (PFS) and the relation between these connections and stuttering severity within the PWS group. Because stuttering is associated with altered activity in multiple brain regions and circuits, we expect our whole-brain analysis to reveal novel connectivity differences related to stuttering and its severity.

## Materials and Methods

### Participants

Twenty persons who stutter (PWS; 5 females, age range: 18–47, median age: 25.5) and 19 PFS (PFS; 4 females, age range: 19–43, median: 24.5) served as controls participated in this study. All participants were right-handed. Potential participants were excluded if they had a history of neurological or motor disorders, were currently on medications with neuropsychological or speech motor effects, or had claustrophobia preventing them from participating in the MRI protocol. The study was approved by COUHES, the institutional review board at MIT.

Participants in the patient group were rated for symptom severity by a speech-language pathologist (DSB) using the Stuttering Severity Instrument-4 (SSI-4; Riley, 2009). Participants were rated based on video, phone, and in-person communication with the speech-language pathologist, who identified the timing and frequency of stuttering events and any accompanying physical characteristics. PWS participants had scores ranging from 13 to 43 (median: 26), representing a wide range of symptom severity at the time of assessment. PFS participants did not have a history of stuttering or other speech disfluencies.

### Data Acquisition

We acquired imaging data at the Athinoula A. Martinos Center for Biomedical Imaging at MIT with a Siemens Magnetom Trio 3-tesla scanner with a 32-channel phased-array head coil. T1-weighted structural images were collected using the magnetization-prepared rapid acquisition gradient echo (MPRAGE) sequence (TR = 2530 ms; TE = 1.64–7.22 ms; TI = 1400 ms; flip angle = 7°; 1 × 1 × 1-mm^3^ isotropic voxels; matrix size: 256 × 256; 172 slices). Whole-brain diffusion-weighted images were collected with a spin-echo echo-planar sequence (TR = 8420 ms; TE = 84 ms; 2 × 2 × 2 mm^3^ isotropic voxels; matrix size: 128 × 128; 67 slices). This included 60 gradient orientations at *b* = 700 s/mm^2^ and 10 no-diffusion images (*b* = 0). Sixty-two volumes of eyes-open resting state data were collected with a 6 s TR. As with the diffusion images, the resting state matrix size was 128 × 128 × 67 with 2 × 2 × 2 mm^3^ isotropic voxels. T1 and diffusion data from these subjects were previously published (Cai et al., 2014). Resting state data were collected from the same subjects in the same MRI sessions.

### Data Processing

Cortical parcellations and subcortical segmentations of the T1-weighted structural images were estimated with FreeSurfer (Fischl, 2012) using the automatic Desikan-Killiany-Tourville (DKT) atlas (Klein and Tourville, 2012; Supplementary Figure 1).

Resting state fMRI data were processed using Nipype (Gorgolewski et al., 2011), a flexible neuroimaging framework that interfaces across multiple software packages. FreeSurfer was used for extracting individual subjects’ ROIs and converting from structural to functional space (Fischl, 2012). Images were registered to a common space using ANTS registration (Avants et al., 2011). Simultaneous motion and slice timing correction was applied (Roche, 2011) and were used to estimate physiological noise with CompCor (Behzadi et al., 2007). Motion outliers were identified with the artifact detection from Nipype and combined with CompCor components and motion parameters for noise reduction. Brain masks were created with the FSL brain extraction tool (Smith, 2002). Data were bandpass-filtered (0.01–0.083 Hz) and smoothed with a 6 mm full-width half-max. (Subcortical data were analyzed without smoothing). For each subject we computed the mean timeseries for each DKT cortical region and FreeSurfer subcortical volume. For each subject we computed the Pearson correlation of each region’s mean timeseries with every other regions’, which were Fisher’s *z*-transformed for comparison across subjects. This ultimately resulted in a symmetrical 84 × 84 connectivity matrix, including 16 subcortical regions. Resting state data were not collected for one PFS subject. A second PFS subject was removed from the analysis after mean activation in the left frontal pole ROI was 0 across all timepoints. One PWS subject was excluded from the resting state analysis due to incomplete whole-brain coverage during the resting scan.

**Figure 1:**
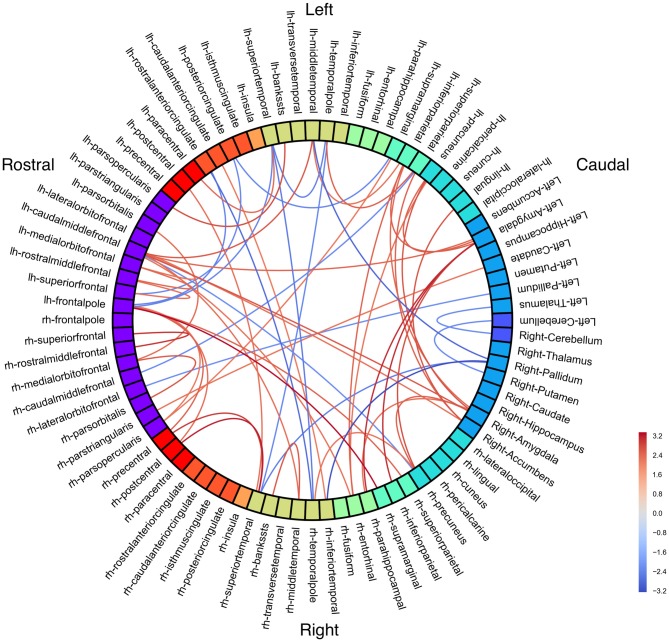
**Resting state group connectivity differences.** Red = people with fluent speech (PFS) > people who stutter (PWS). Blue = PWS > PFS. Color represents Student’s *t*-statistic. All connections *p* < 0.033 (uncorrected).

Diffusion-weighted images were processed with TRACULA (Yendiki et al., 2011), which applies the ball-and-stick model (FSL’s *bedpostx*) for probabilistic tractography of known white matter pathways using anatomically constrained priors from FreeSurfer. We then performed local probabilistic tractography with *probtrackx2* (Behrens et al., 2007) based on *bedpostx* outputs. This was performed between all parcellations and segmentations from FreeSurfer, extended 2 mm into white matter and registered to each each subject’s diffusion space. The number of connections for a given seed region to a target region were normalized by dividing out the total number of tracks from the seed region. This resulted in an asymmetrical 89 × 89 connectivity matrix, including 21 subcortical regions. (See Figure 1 for region names). However, since probabilistic tractography has no information regarding the direction of these connections, we averaged the *a* → *b* and the *b* → *a* normalized track counts to create a symmetrical connectivity matrix.

### Statistical Data Analysis

Differences in structural connectivity between PWS and PFS were computed with non-parametric Wilcoxon rank-sum test for each region × region connectivity measure. Functional regional connectivity group differences were compared using independent two-sample *t*-tests. Relationships between connectivity and SSI-4 (stuttering symptom severity) were determined with the Pearson correlation coefficient. All tests resulted in two-tailed *p*-values. False discovery rate (FDR) was used to test for multiple comparisons.

Regional network strength for probabilistic tractography analysis was computed as the sum of all connections from a given region to all other regions.

## Results

### Resting State Connectivity: Group Differences

To examine how stuttering may affect functional co-activation of regions across the whole brain, we measured Blood-oxygen-level dependent (BOLD) activity during a resting state fMRI paradigm and compared functional connectivity results between the PWS and PFS groups. No connections were significant with an FDR-corrected threshold of *p* < 0.05. With an uncorrected threshold of *p* < 0.033 (Figure 1; see Supplementary Figure 2 for unthresholded results), PWS had stronger subcortical connections between right cerebellum and left thalamus, as well as between right putamen and left cerebellum and between right pallidum and left middle temporal gyrus, right superior, and right inferior temporal gyrus. Bilateral amygdala had decreased connectivity with right precuneus and parahippocampal cortex in PWS.

In the speech network, left superior temporal gyrus is less connected to left paracentral lobule, but more connected to bilateral temporal pole in PWS. Left pars opercularis is less connected with left superior temporal sulcus in PWS, while right pars opercularis is less connected with left supramarginal gyrus.

Other left hemisphere regions with large connectivity differences include frontal pole and caudal middle frontal gyrus.

### Resting State Connectivity: Stuttering Symptom Correlations

**Figure 2:**
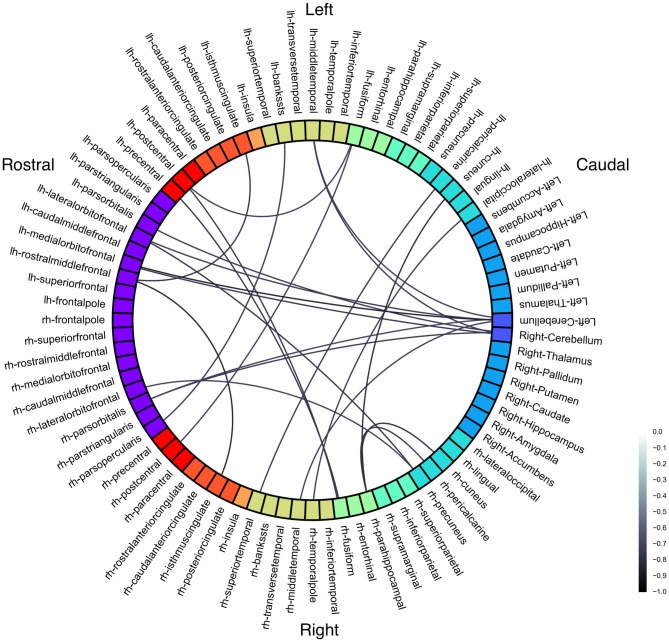
**Functional connectivity correlations with Stuttering Severity Instrument 4 (SSI-4).** Color represents Pearson’s correlation coefficient (*r*). All connections *p* < 0.001 (uncorrected).

We next investigated how functional connectivity varied in relation to stuttering symptom severity. Correlations between stuttering symptoms and connectivity may highlight compensatory connectivity patterns in less symptomatic PWS or dysfunctional connections in more severe stutterers. We looked within the stuttering group only and measured correlations between regional connectivity patterns and the stuttering severity scores (SSI-4) of PWS participants (Figure 2; see Supplementary Figure 3 for unthresholded results). All significant resting state connectivity correlations with SSI-4 were negative.

**Figure 3:**
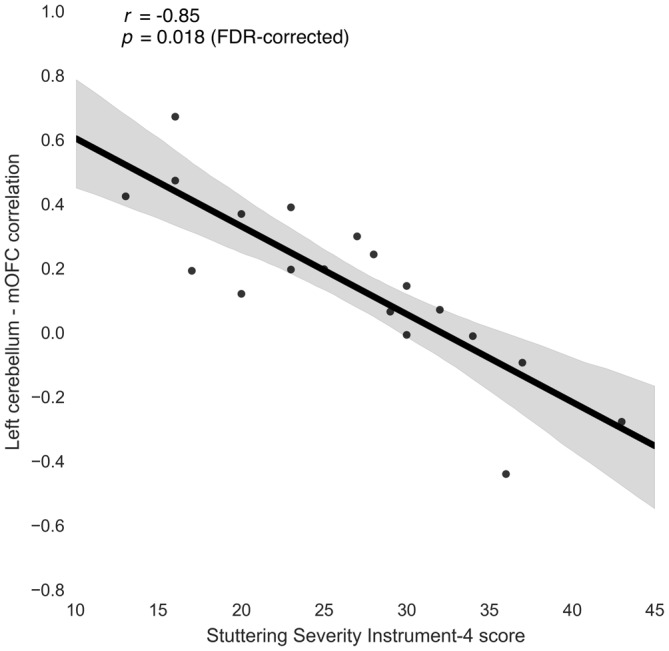
**Relationship between stuttering severity instrument-4 (SSI-4) and left cerebellum-medial orbitofrontal cortex (mOFC) functional connectivity in people who stutter**.

Three connections were significant after correcting for multiple comparisons (FDR-corrected *p* < 0.05, uncorrected *p* < 10^−5^): left cerebellum to left medial orbitofrontal (*r* = −0.85; Figure 3), left rostral middle frontal to right isthmus cingulate (*r* = −0.83), and left cuneus to right parahippocampal (*r* = −0.80). The correlations between left rostral middle frontal cortex and left isthmus cingulate (*r* = −0.76) and between right cuneus and right parahippocampal (*r* = −0.78) were similar but did not survive FDR correction (uncorrected *p* < 5 × 10^−4^).

To check whether these correlations were due to extremely high or extremely low connectivity in the PWS group as a whole, we compared the connectivity measures for these connections between PWS and the PFS control participants. The left cerebellum-medial orbitofrontal connection was slightly stronger on average in PWS compared to PFS, but this difference was not significant (*t* = 1.26, uncorrected *p* = 0.217). The group differences between left rostral middle frontal cortex and bilateral isthmus cingulate were also not significant (*t* < 0.5, uncorrected *p* > 0.65).

At a slightly less stringent threshold (uncorrected *p* < 0.001), we find stuttering severity is anticorrelated with functional connectivity between bilateral cerebellum and left frontal cortex; left superior temporal sulcus and right pars opercularis; left fusiform with bilateral postcentral gyrus; right fusiform with left precentral and postcentral gyri; and right precuneus with left right lateral OFC. Cerebellum has decreased connectivity with bilateral middle temporal gyrus, left orbitofrontal (pars orbitalis, lateral orbitofrontal, and medial orbitofrontal) cortex, and right pars orbitalis.

Despite the group differences in frontal-temporal-amygdalar connectivity, there were no amygdalar connectivity correlations with SSI.

### White Matter Connectivity: Regional Network Strength

In addition to functional connectivity measures, we can also estimate structural connectivity between regions across the whole brain. Using probabilistic tractography of diffusion-weighted MRI, we inferred how each region is physically connected to all other regions via white matter connections.

With this tractography method, we first investigated the total number of white matter streamlines (connections) to each region, revealing the graph theory measure known as regional network strength (Table 1). No measures were significant with an FDR-corrected threshold of *p* < 0.05. Comparing groups, left pars triangularis had significantly greater network strength in PFS vs. PWS (*t* = 2.79, uncorrected *p* = 0.008). Left lateral OFC had the second greatest difference between groups (*t* = 2.03, uncorrected *p* = 0.05).

**Table 1:** **White matter network strength by region of interest (ROI)**.

PFS > PWS group differences		
*t* statistic	*p* value (uncorrected)	Region of interest
2.79	0.008	**Left pars triangularis
2.03	0.050	Left lateral orbitofrontal

**PWS ROI strength correlations with SSI-4**		

**Pearson *r***	***p* value (uncorrected)**	**Region of interest**

−0.47	0.035	Left pericalcarine
−0.46	0.041	Left precuneus
−0.62	0.004	**Left superior parietal
−0.51	0.020	Left transverse temporal
−0.50	0.025	Left superior temporal sulcus
−0.50	0.026	Left superior temporal gyrus
−0.45	0.044	Left isthmus cingulate
−0.47	0.035	Left paracentral
−0.50	0.025	Left precentral
−0.56	0.011	Left pars orbitalis
−0.47	0.038	Left caudal middle frontal
−0.49	0.029	Left superior frontal
−0.49	0.030	Right insula
−0.46	0.042	Right superior temporal gyrus
−0.62	0.003	**Right temporal pole
−0.48	0.032	Right precuneus
−0.44	0.050	Right putamen
−0.56	0.010	**Right cerebellum

Top: group differences between people with fluent speech (PFS) and people who stutter (PWS). Bottom: correlations with Stuttering Severity Instrument 4 (SSI-4) in PWS. **Significant at p < 0.01 (uncorrected).

Within the stuttering group, left superior parietal cortex and right temporal pole network strength were strongly negatively correlated with stuttering symptom severity (*r* = −0.62, uncorrected *p* < 0.004). Other regions with strong negative correlations (*r* < −0.50, uncorrected *p* < 0.03) were left Heschl’s gyrus, superior temporal sulcus, superior temporal gyrus, precentral gyrus, and pars orbitalis, and right cerebellum.

### White Matter Connectivity: Group Differences

**Figure 4:**
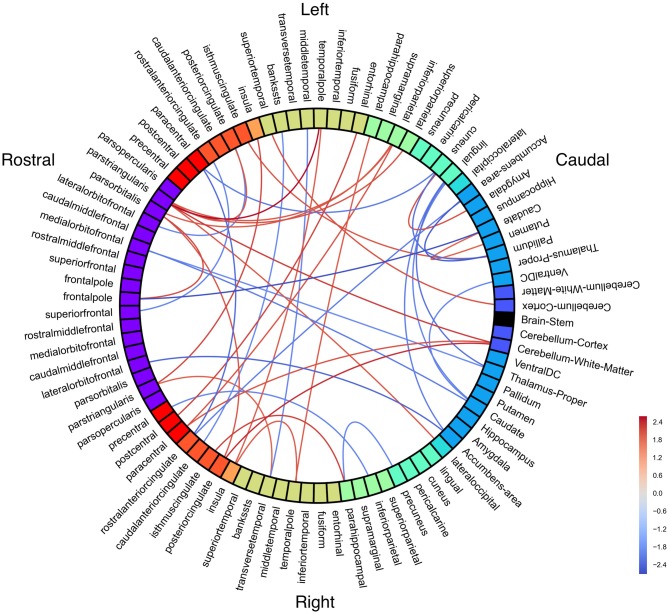
**White matter connectivity differences between groups.** Red = people with fluent speech (PFS) > people who stutter (PWS). Blue = PWS > PFS. All connections *p* < 0.05 (uncorrected).

We next examined probabilistic tractography between all cortical and subcortical regions to investigate whole-brain white matter connectivity differences between PWS and fluent-speaking controls. No connections were significant with an FDR-corrected threshold of *p* < 0.05. Results are summarized in Figure 4.

Notably, right cerebellum was significantly less connected with left pars triangularis, right paracentral lobule, and right posterior cingulate in PWS than in PFS. Left pars triangularis had the greatest number of significant connection differences between groups, with all connections being weaker in PWS.

### White Matter Connectivity: Symptom Severity Correlations

**Figure 5:**
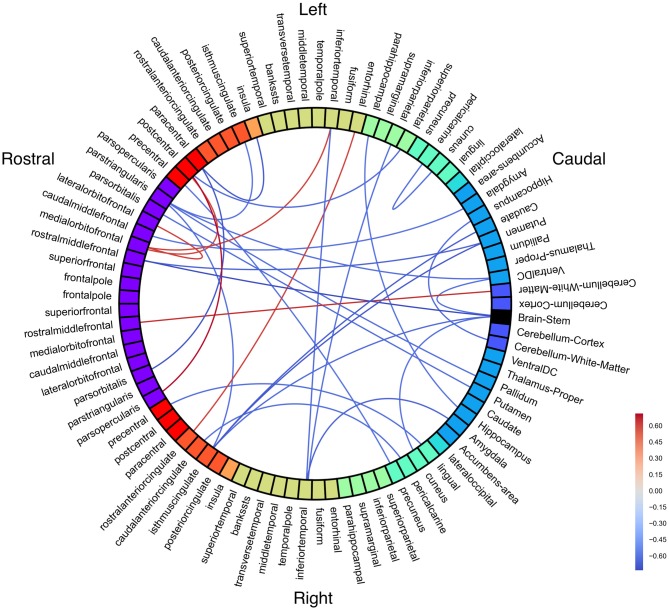
**White matter connectivity correlated with Stuttering Severity Instrument 4 (SSI-4) in people who stutter (PWS).** All connections *p* < 0.01 (uncorrected).

Structural connectivity may vary within the PWS group as a function of stuttering severity. No connections were significant with an FDR-corrected threshold of *p* < 0.05. Probabilistic tractography connectivity correlations with SSI-4 were largely negative, including left pars triangularis and the brainstem as hubs of strong anticorrelations with severity (Figure 5).

Positive correlations with SSI-4 include left postcentral gyrus with left medial OFC and right pars opercularis. Left medial OFC was a hub of positive correlations with stuttering severity.

### White Matter Tract Analysis

Whereas the previous analyses investigated region-to-region structural connectivity via individual streamlines, we can also use our knowledge of the anatomy of large white matter bundles to examine differences in major tracts that are associated with stuttering. Based on major white matter tract reconstruction with TRACULA, we found that PWS (vs. PFS controls) had a larger left uncinate volume (*t* = 2.39, uncorrected *p* = 0.022), greater posterior corpus callosum length (*t* = 2.65, uncorrected *p* = 0.012), and lower mean FA in the right parietal tract of the superior longitudinal fasciculus (SLFP; *t* = 2.11, uncorrected *p* = 0.042). Right SLFP has decreased FA compared to the left SLFP in PWS but not PFS (*t* = 2.37, uncorrected *p* = 0.023).

Average FA in the right SLFP is negatively correlated with stuttering symptom severity (*r* = −0.482, uncorrected *p* = 0.032). Lengths of the left SLFP (*r* = 0.478, uncorrected *p* = 0.033) and left anterior thalamic radiation (*r* = 0.539, uncorrected *p* = 0.014) were positively correlated with SSI.

No major tract group differences or correlations were significant with an FDR-corrected threshold of *p* < 0.05.

## Discussion

Using resting state and diffusion MRI, we found that people who stutter had increased functional and structural connectivity between the cerebellum, midbrain, and thalamus compared to PFS. However, in individuals with the greatest stuttering severity, the subcortical network had reduced connectivity with frontal cortical regions than in individuals with fewer stuttering symptoms, suggesting that PWS may be able to compensate for a dysfunctional basal ganglia-thalamocortical (BGTC) cortical network by relying on the cerebellum and OFC.

Our findings support the hypothesis that both cerebellum and OFC are involved in successful compensation for stuttering symptoms and suggest that the best compensation occurs when the two compensatory networks—subcortical (cerebellar) and cortical (orbitofrontal)—are synchronized. Cerebellar connections—largely functional connectivity with left OFC—were strongly negatively correlated with stuttering severity. Similarly, both left pars orbitalis and right cerebellum white matter network strength were significantly negatively correlated with stuttering severity.

In the typically functioning brain, the cerebellum compares the predicted sensory outcomes of an action to the actual sensory consequences (Blakemore et al., 2001), with larger neural responses occurring when feedback has been experimentally altered (Brooks et al., 2015). In particular, cerebellar monitoring appears to be an increase in the function of attention as opposed to an automatic monitoring process (Allen et al., 1997). Cerebellar damage is linked to impaired internal predictions for motor responses, at least in the visual domain (Therrien and Bastian, 2015), and individuals with spinocerebellar ataxia are likely to have difficulty with auditory integration and temporal gap detection (Zeigelboim et al., 2015).

In people who stutter, multiple studies have found increased cerebellar activity following speech fluency training (De Nil et al., 2001; Lu et al., 2012). OFC has been implicated in stuttering symptom avoidance (Sowman et al., 2012) and recovery (Kell et al., 2009), especially in the left hemisphere. We report here for the first time that these regions are part of functionally and structurally connected circuits associated with compensation and symptom avoidance.

Deficiencies in basal ganglia function, particularly of the BGTC circuit (Alm, 2004; Craig-McQuaide et al., 2014), are hypothesized to underlie stuttering symptomatology. We found stronger functional connectivity between right pallidum and bilateral temporal cortices in people who stutter, as well stronger structural connectivity including connections from left pallidum, left ventral DC, right thalamus, bilateral caudate, and bilateral nucleus accumbens. Left putamen resting connectivity with caudal ACC was lowest in subjects with the most severe stuttering symptoms, as was left putamen and caudate structural connectivity with isthmus cingulate. Hyperactivity in the basal ganglia could disinhibit speech motor commands in people who stutter, resulting in speech disfluencies. Indeed, caudate activity is correlated with stuttering severity, and it can be mitigated with therapy (Giraud et al., 2008). Meanwhile, putamen overactivity may function a compensatory mechanism in stuttering (Neumann et al., 2003). These findings suggest that basal ganglia dysfunction is involved in stuttering, and that at least some of the striatal connections contribute to successful compensation for symptoms as opposed to underlying the disorder.

Studies involving people who stutter have typically focused on the cortical speech network. We also found connectivity abnormalities in the left hemisphere perisylvian speech network. Pars opercularis in the inferior frontal gyrus was functionally less connected with left superior temporal sulcus in PWS than in PFS, but it was functionally more connected with right temporal pole. Pars triangularis had significantly decreased network connectivity strength between PFS and PWS. Network strength was negatively correlated with stuttering severity in left precentral, paracentral, superior temporal sulcus, and bilateral superior temporal gyrus. Taken together, these support the theory of a weaker feedforward network involving inferior frontal and precentral gyri, with compensation provided by a feedback network involving motor and temporal regions (Tourville and Guenther, 2011).

These results fall in line with previous evidence from task-based fMRI, structural gray matter analysis, white matter diffusion analysis, and white matter connectivity analysis (for a recent review, see Craig-McQuaide et al., 2014). Jäncke et al. (2004) found increased white matter volume in the right hemisphere using voxel-based morphometry (VBM). Others have found decreased white matter integrity along the superior longitudinal fasciculus in the left hemisphere, a tract known to connect the auditory, motor, and planning regions crucial for speech production (Sommer et al., 2002; Watkins et al., 2008). While numerous studies have shown decreased FA in speech motor areas in PWS, there is little consistency in where these differences are focused, although a few studies have shown approximately similar locations of FA differences in the posterior arcuate fasciculus (Sommer et al., 2002; Chang et al., 2008; Watkins et al., 2008; Connally et al., 2014; Cai et al., 2014). This, along with the present connectivity analysis and that of Cai et al. (2014), suggest that it is connectivity between regions (rather than white matter integrity in a given location) that is impaired in persistent developmental stuttering.

However, although we did find left hemisphere connectivity differences consistent with previous studies, we did not fully replicate known stuttering dysfunctions in the literature. For instance, we did not find underconnectivity in left premotor and primary motor cortex, as has been described previously in PWS (Cai et al., 2014). These differences may arise from the parcellations used to map the cortex. The DKT atlas used in the present study is based on gross anatomical landmarks, creating broadly defined regions that often combine regions with distinct functions but indistinct anatomical boundaries. As a result, regions like primary motor cortex and premotor cortex are lumped together into “precentral.” Thus, while group connectivity differences in some regions are similar between our analysis and that of Cai et al. (2014)—such as stronger diffusion connectivity in PFS between left pars triangularis and pars orbitalis—we were not equipped to replicate their findings of stronger ventral premotor cortex—ventral somatosensory cortex connections in PFS compared to PWS.

In sum, our cerebellar-orbitofrontal results extend previous findings in the literature, while our basal ganglia and cortical speech network results fall in line with those from previous studies. Other interesting findings from the current study have less support in the literature. Negative correlations between functional connectivity and stuttering symptom severity between bilateral fusiform gyrus and bilateral postcentral gyrus suggest a unique role for the fusiform gyrus in stuttering, which has been observed (Brown et al., 2005) but not explained previously.

While these findings occur outside of the traditionally cited speech-related regions, recent neuroimaging work in stuttering has focused primarily on the cortical speech network. For instance, Cai et al. (2014) performed diffusion tractography on the same group of subjects as in the present study using a speech-specific cortical atlas. Chang and Zhu (2013) restricted functional and structural connectivity analyses to speech production-related structures, although other work has looked at fractional anisotropy differences throughout the brain (including increased FA in the cerebellum; Chang et al., 2015).

Another strength of the present study is in incorporating both structural and functional connectivity measures across the whole brain in the same group of subjects. For example, in both the structural and functional data, subcortical connectivity group differences tended to be stronger in PWS than in the PFS controls. Indeed, structural connectivity networks largely underlie their functional counterparts (Sporns, 2011). However, because functional correlations can be driven by indirect structural connections, one cannot infer structural connectivity from a functional network. Indeed, while the strongest (anti-)correlation with stuttering symptom severity was the connection between OFC and cerebellum, there are no known direct anatomical connections between prefrontal cortex and cerebellum, and fewer than 1% of thalamic neurons reaching OFC originated in areas of the thalamus connected to cerebellum (although 23% of connections are in the area of connections to the dopaminergic substantia nigra; see Middleton and Strick, 2001).

Our study represents the first investigation of functional connectivity in English-speaking adults who stutter. These differences reflect a combination of the traits underlying the disorder itself as well as the result of decades of stuttering (and compensation) experience. Fully teasing apart these two contributing factors will require longitudinal developmental and brain imaging data, which do not yet exist.

Technical limitations add an additional challenge to the goal of uniting structural and functional connectivity networks. For instance, the low temporal resolution of fMRI results in a limited frequency range of functional oscillations. While previous research has shown that a longer TR is sufficient for recording resting state BOLD activity (Van Dijk et al., 2010), it is possible that some meaningful signal will be excluded based on these methods. Meanwhile, diffusion tractography has difficulty resolving complex fiber crossings or sharp turns in streamlines. As a result, the cerebral peduncle can interrupt streamlines from the cerebellum, and the corticospinal/corticobulbar tracts may be missing ventral sensorimotor projections. Thus, while both structural and functional connectivity can and do yield instructive insight into brain differences between people who stutter and people with normal speech, caution should be exercised in synthesizing and interpreting the results from each.

Persistent developmental stuttering is a common speech fluency disorder that can seriously impede an individual’s ability to communicate. Nonetheless, some people who stutter develop compensatory speech techniques to improve their fluency, minimizing the effects of stuttering symptoms in their daily communication. Previous research identified two separate brain regions—OFC and the cerebellum—that may be linked to compensation for stuttering symptoms. In this study, we show that stronger functional connections between these anatomically distal regions are correlated with decreased stuttering symptom severity in people who stutter, suggesting that synchrony between these cortical and subcortical regions may enable the most successful compensation for stuttering symptoms.

## Ethics Statement

This research was approved by the Committee on the Use of Humans as Experimental Subjects (COUHES) at MIT.

## Author Contributions

SC and SSG collected the data. KRS processed and analyzed the data and wrote the first draft of the manuscript. SC, DSB, JSP, FHG, and SSG revised the manuscript.

## Funding

This work was supported by National Institutes of Health grants R01-DC007683 (PI: FHG), R56-DC0010849 (PI: JSP), and T32-DC000038 (trainee: KRS).

## Conflict of Interest Statement

The authors declare that the research was conducted in the absence of any commercial or financial relationships that could be construed as a potential conflict of interest.

## Supplementary Material

Supplementary Figure 1 **Structural parcellation of the cortex based on the Desikan-Killiany-Tourville parcellations of Mindboggle-101 data (Klein and Tourville, 2012). Figure adapted from Klein and Tourville (2012)**.

Supplementary Figure 2 **Functional connectivity matrix—significant group differences between people who stutter and people with fluent speech**.

Supplementary Figure 3 **Functional connectivity matrix—correlations with Stuttering Symptom Instrument-4 (SSI-4)**.
