# Supplementary figures and images for: Decreased Cerebellar-Orbitofrontal Connectivity Correlates with Stuttering Severity: Whole-Brain Functional and Structural Connectivity Associations with Persistent Developmental Stuttering
Source: Front Hum Neurosci. 2016 May 3;10:190. doi: 10.3389/fnhum.2016.00190 (PMC4855981; doi:10.3389/fnhum.2016.00190)

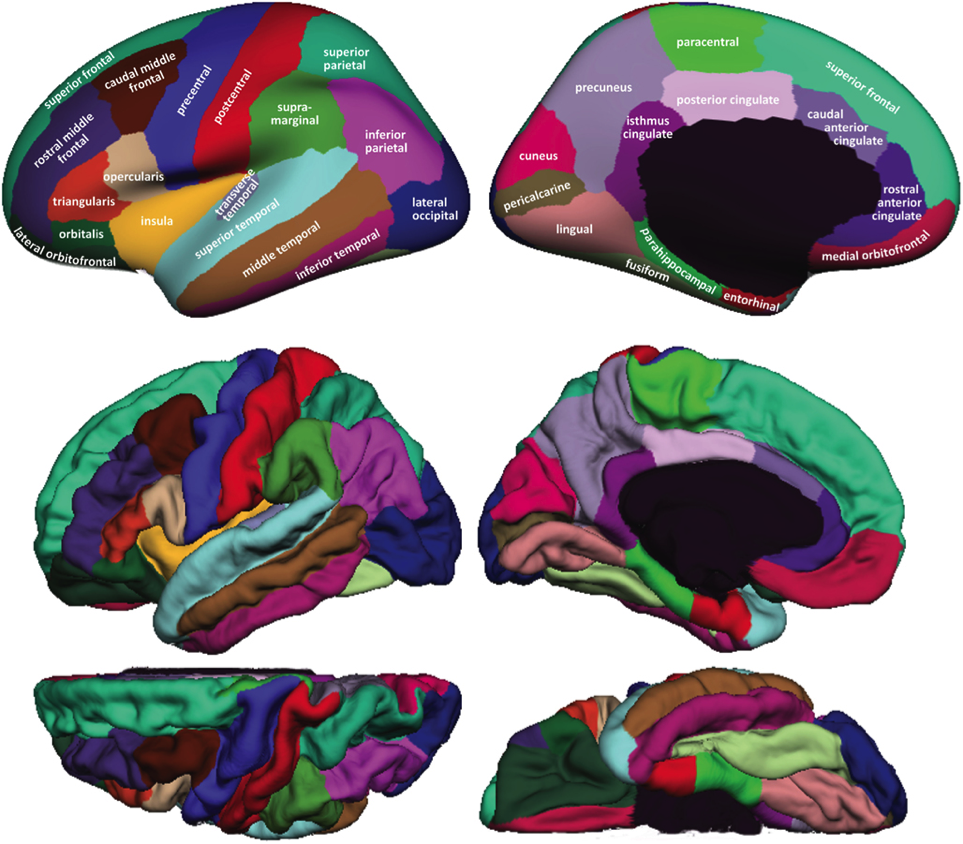

Supplement: Supplementary Figure 1 — Structural parcellation of the cortex based on the Desikan-Killiany-Tourville parcellations of Mindboggle-101 data (Klein and Tourville, 2012). Figure adapted from Klein and Tourville (2012). [file Image_1.png]

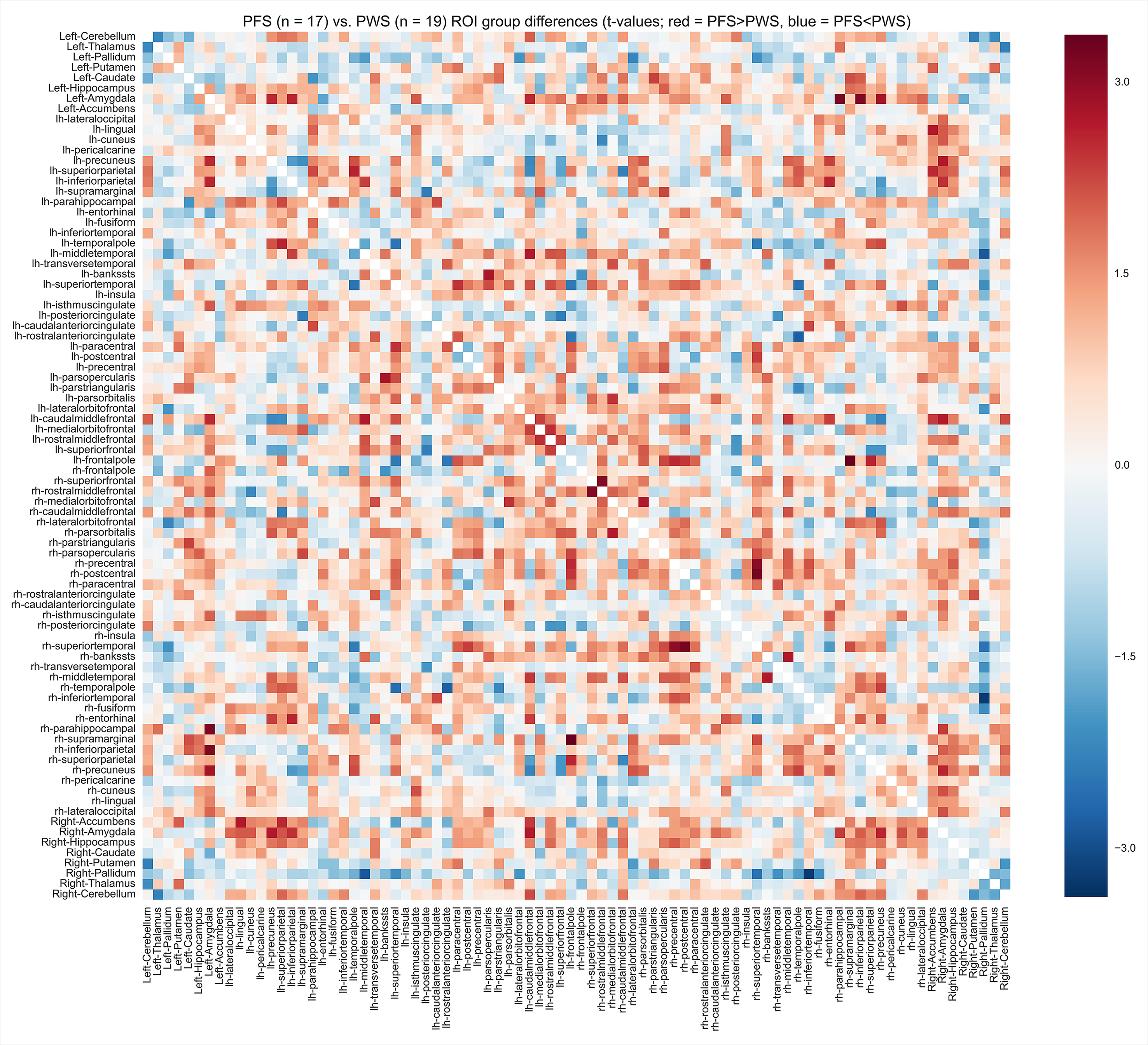

Supplement: Supplementary Figure 2 — Functional connectivity matrix—significant group differences between people who stutter and people with fluent speech. [file Image_2.tiff]

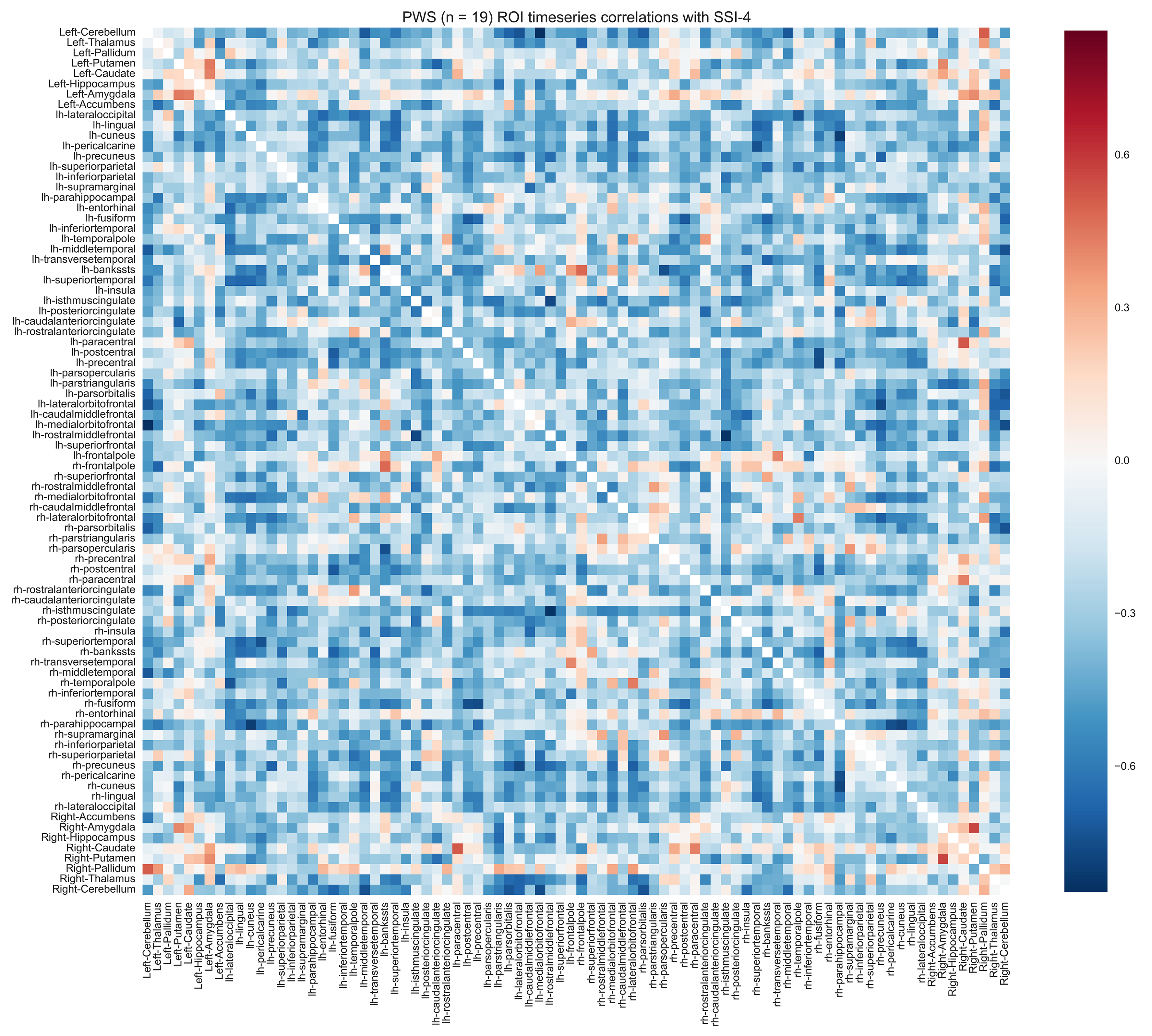

Supplement: Supplementary Figure 3 — Functional connectivity matrix—correlations with Stuttering Symptom Instrument-4 (SSI-4). [file Image_3.tiff]
